# Designing and conducting a cluster-randomized trial of ICU admission for the elderly patients: the ICE-CUB 2 study

**DOI:** 10.1186/s13613-016-0161-5

**Published:** 2016-07-29

**Authors:** Ariane Boumendil, Maguy Woimant, Jean-Pierre Quenot, François-Xavier Rooryck, Foued Makhlouf, Youri Yordanov, Samuel Delerme, Khalil Takun, Patrick Ray, Marie-Clément Kouka, Claire Poly, Maité Garrouste-Orgeas, Caroline Thomas, Tabasome Simon, Sylvie Azerad, Guillaume Leblanc, Dominique Pateron, Bertrand Guidet, on behalf of the ICE-CUB 2 study network, Bertrand Guidet, Dominique Pateron, Youri Yordanov, Erwan Debuc, Didier Dreyfuss, Jean-Damien Ricard, Patrick Brun, Christophe Leroy, Yves Cohen, Frédéric Adnet, Maguy Woimant, Jean-Paul Mira, Benoit Doumenc, Khalil Takun, Thomas Similowski, Bruno Riou, Samuel Delerme, Jean-Pierre Quenot, Didier Honnart, Jean-François Timsit, Pierrick Gueringuerin, Françoise Guerin, Maxime Maignan, Foued Makhlouf, Jean-François Poussel, François Braun, François Fourrier, Patrick Goldstein, Pierre Williatte, Pierre Gosselin, Eric Wiel, Francesco Santoli, Pierre Charestan, Claire Poly, Hervé Mentec, Catherine Le Gall, Karima Sahraoui, Christophe Baillard, Loïc Pontual, Nicolas Javaud, Benoît Misset, Maité Garrouste-Orgeas, Olivier Ganansia, François-Xavier Rooryck, Jean Luc Aim, Abudlrazak El Rifai, François-Xavier Rooryck, Jean Reignier, Laurent Martin-Lefevre, Philippe Fradin, Claire Mauriat, Emelyne Cwicklinski, Hervé Dupont, Michel Slama, Christine Ammirati, Justine Gallou, Muriel Fartoukh, Michel Djibre, Patrick Ray, Edwin Rouff, Bertrand Souweine, Ali Ait Hssain, Jeannot Schmidt, Daniel Pic, Farès Moustafa, Alain Mercat, Nicolas Lerolle, Pierre-Marie Roy, Frédéric Baud, Patrick Plaisance, Sophie Montagnon, Bertrand Galichon, Michel Wolff, Bruno Mourvillier, Enrique Casalino, Christophe Choquet, Bernard, Gaëlle Juillien, Jean-Yves Fagon, Emmanuel Guerot, Philippe Juvin, Anabela Patzak, Bruno Verdiere, Vincent Ioos, Marie-clément Kouka, Audrey Berthoumieu, Christian Richard, Nadia Anguel, Raphael Maurice, Sophie Sarnel, Stéphane Diez, Antoine Vieillard Baron, Sébastien Beaune, Julie Grenet

**Affiliations:** 1 Hôpital Saint-Antoine, Service de Réanimation Médicale (Intensive Care Unit - ICU), Assistance Publique - Hôpitaux de Paris (AP-HP), 184 rue du Faubourg Saint-Antoine, 75012 Paris, France; 2 Hôpital Avicenne, Service d’Accueil des Urgences (SAU, Emergency Department), AP-HP, 93009 Bobigny, France; 3 ICU, CHU de Dijon, 21079 Dijon, France; 4 SAU, Hôpital Saint-Joseph, 75014 Paris, France; 5 SAU, CHU de Grenoble, 38700 Grenoble, France; 6 Hôpital Saint-Antoine, SAU, AP-HP, 75012 Paris, France; 7 Hôpital Pitié Salpétrière, SAU, AP-HP, 75013 Paris, France; 8 Hôpital Cochin, SAU, AP-HP, 75014 Paris, France; 9 Hôpital Tenon, SAU, AP-HP, 75020 Paris, France; 10 SAU, Hôpital Delafontaine, 93200 Saint Denis, France; 11 SAU, Hôpital Robert Ballanger, 93602 Aulnay-Sous-Bois, France; 12 ICU, Hôpital Saint Joseph, 75014 Paris, France; 13 Hôpital Saint-Antoine, Acute Geriatric Ward, AP-HP, 75012 Paris, France; 14 Hôpital Saint-Antoine, URC Est, AP-HP, 75012 Paris, France; 15 Hôpital Ambroise Paré, URC Ouest, AP-HP, 92104 Boulogne-Billancourt, Paris, France; 16 Department of Anesthesiology and Critical Care, Université Laval, Québec, QC Canada; 17 UPMC Univ Paris 06, Sorbonne Universités, Paris, France; 18 UMR_S 1136, Institut Pierre Louis d’Épidémiologie et de Santé Publique, INSERM, 75013 Paris, France

**Keywords:** Elderly, Intensive care, Ethics

## Abstract

**Background:**

The benefit of ICU admission for elderly patients remains controversial. This report highlights the methodology, the feasibility of and the ethical and logistical constraints in designing and conducting a cluster-randomized trial of intensive care unit (ICU) admission for critically ill elderly patients.

**Methods:**

We designed an interventional open-label cluster-randomized controlled trial in 24 centres in France. Clusters were healthcare centres with at least one emergency department (ED) and one ICU. Healthcare centres were randomly assigned either to recommend a systematic ICU admission (intervention group) or to follow standard practices regarding ICU admission (control group). Clusters were stratified by the number of ED annual visits (<44,616 or >44,616 visits), the presence or absence of a geriatric ward and the geographical area (Paris area vs other regions in France). All elderly patients (≥75 years of age) who got to the ED were assessed for eligibility. Patients were included if they had one of the pre-established critical conditions, a preserved functional status as assessed by an ADL scale ≥4 (0 = very dependent, 6 = independent), a preserved nutritional status (subjectively assessed by physicians) and without active cancer. Exclusion criteria were an ED stay >24 h, a secondary referral to the ED and refusal to participate. The primary outcome was the mortality at 6 months calculated at the individual patient level. Secondary outcomes were ICU and hospital mortality, as well as ADL scale and quality of life (as assessed by the SF-12 Health Survey) at 6 months.

**Results:**

Between January 2012 and April 2015, 3036 patients were included in the trial, 1518 patients in 11 clusters allocated to intervention group and 1518 patients in 13 clusters allocated to standard care. There were 51 protocol violations.

**Conclusions:**

The ICE-CUB 2 trial was deemed feasible and ethically acceptable. The ICE-CUB 2 trial will be the first cluster-randomized trial to assess the benefits of ICU admission for selected elderly patients on long-term mortality.

*Trial registration* Clinical trials.gov identifier: NCT01508819

**Electronic supplementary material:**

The online version of this article (doi:10.1186/s13613-016-0161-5) contains supplementary material, which is available to authorized users.

## Background

The ageing of the population leads to an increase in intensive care unit (ICU) admissions among elderly patients [1], and patients over 80 years represent 10–20 % of all ICU admissions in Western countries [2–5]. Elderly patients are more vulnerable to acute stress due to age-related diminution of physiological reserve and more common frailty than younger patients [6]. This vulnerability of elderly patients to acute stress makes the benefit of an ICU admission uncertain. Because a judicious resources use is a source of concern in the ICU [7], only patients who would benefit from an ICU stay should be admitted [8]. However, there are no clear recommendations to help physicians in the ICU admission decision-making process for elderly patients [9]. The absence of recommendations leads to an heterogeneity in clinical practices within the same region and across different countries [10, 11].

Until now, there is no randomized clinical trial of ICU admission for elderly patients in the literature. Moreover, epidemiological and observational studies [2, 5, 9, 10, 12–16] failed to provide clear evidence of benefit. In a prospective, observational, cohort study (ICE-CUB 1 study, *n* = 2646 patients) [10], ICU admission for critically ill elderly patients did not affect the mortality at 6 months (50.6 % for patients admitted to the ICU compared to 50.7 % for all other elderly patients) [10, 17–19]. However, preserved functional status, preserved nutritional status and absence of cancer were associated with a better prognosis at 6 months (62 % mortality for patients without any of these factors versus 31 % mortality for patients with at least one) [10].

Past studies demonstrated a great variation in ICU admission rates for elderly patients, ranging from 8 to 40 % [4, 10, 12–14, 20]. This variability is in part due to differences in medical practices regarding ICU admission, differences in local policies and variability of ICU beds availability. The interpretation of these studies is limited by the absence of consideration of the triage process by the ED physicians prior to the ICU admission. If ED physicians are very selective for ICU candidates, this will result in ICU admission requests for highly selected patients only, then a low refusal rate by intensive care physicians. On the other hand, a more liberal process for ICU admission will result in a higher refusal rate by intensive care physicians. In the ICE-CUB 1 study, only 25 % of patients with a critical condition were referred to the ICU by the ED physicians [10]. Independent factors associated with the absence of ICU referral by the ED physicians were a high age, an active cancer, a low severity of the acute illness and a low score on Activities of Daily Living scale (ADL scale) [11]. Therefore, to better understand the ICU admission decision-making process for the elderly patients, there is a need to evaluate both the ED and ICU physician triage processes.

This report highlights the methodology, the feasibility of and the logistical and ethical constraints in designing and conducting a study to assess the benefit of ICU admission for critically ill elderly patients (the ICE-CUB 2 study).

## Methods

### Objective and design

We aimed at designing a feasible, ethically acceptable, generalizable and reproducible trial with relevant outcomes. Since randomization of ICU admission at individual patient level is considered unethical (by virtue of beneficence, non-maleficence and respect of the patient’s autonomy), we designed an interventional open-label cluster-randomized trial. Our primary research question was whether a recommendation for a systematic ICU admission for critically ill elderly patients who got to the ED can improve survival at 6 months, compared to usual care.

### Participating hospitals

To maximize the generalizability of the results, the ICE-CUB 2 study aimed to involve a geographically and clinically diverse spectrum of EDs and ICUs across France. Clusters were academic or non-academic healthcare centres with at least one ED and one ICU willing to participate.

### Patients

In each participating hospital, all elderly patients (>75 years of age) who got to the ED were assessed for eligibility. Patients were included in the trial if they met all the inclusion criteria and no exclusion criteria.

*Inclusion criteria*

1. A diagnosis among a pre-established list of critical conditions (Table 1).

**Table 1:** Main admission criteria

Cardiology	Cardiogenic shock
	Cardiac insufficiency requiring NIV
	Severe cardiac rhythm abnormalities
Surgery	Neurosurgery
	Surgery for poly-traumatism
	Cardiac surgery
	Digestive surgery
	Surgery other
Coma	Coma—metabolic
	Coma—toxic
	Coma—stroke
	Coma—status epilepticus
	Coma—traumatism
	Coma—anoxic
	Coma—cerebral hypertension
Respiratory	Acute respiratory failure with COPD
	Pulmonary embolism
	Bilateral pneumonia
	Acute respiratory failure requiring tracheal intubation
	Acute respiratory failure requiring NIV
	Acute respiratory failure requiring active physiotherapy
GI	GI tract haemorrhage
	Pancreatitis
	Acute liver insufficiency
	Abdominal emergency
Shock	Septic shock
	Haemorrhagic shock
	Hypovolemic shock
	Shock others
Renal	Acute kidney failure
Polytraumatism	
Miscellaneous	Miscellaneous
2. A preserved functional status, as assessed by an ADL scale [21] ≥4 (0 = very dependent, 6 = independent).
3. A preserved nutritional status (defined as the absence of cachexia).
4. No known active cancer.

*Exclusion criteria*

1. An emergency department stay >24 h.
2. A secondary referral to the emergency department.
3. Patient’s or surrogate decision-makers’ refusal to participate.
4. No social security coverage.

The list of critical conditions that required ICU admission was retrieved from the ICE-CUB 1 study [11]. This list of critical conditions adapted to the elderly patient was established by a Delphi consensus method among emergency physicians and adapted from the *Guidelines for intensive care unit admission, discharge, andtriage* [22]. We also restricted the list mostly to critical conditions that potentially require an organ support (Table 1). In order to focus on patients perceived as good candidates for ICU admission, we excluded patients with factors of poor prognosis, as identified in the ICE-CUB 1 study: presence of cachexia, active cancer and a decline in functional status [10]. We used the ADL scale [21] to evaluate the functional status, since this scale is widely employed and easy to use. We subjectively assessed the nutritional status by physician at bedside because it is faster and easier compared to a BMI calculation in an emergency room setting and because nutritional laboratory assessments are not reliable in critically ill patients [23].

### Randomization

The allocation schedule was independently established by a statistician (AB) at the clinical research unit using a computer-generated randomization list. Randomization was stratified by the annual number of ED visits (the cut-off value was the median of the annual number of ED visits in each participating centres, *N* = 44,616) and the presence or absence of a geriatric ward and the geographical area (Paris area vs other regions in France). The allocation was kept concealed by the clinical research unit until the beginning of the study. The investigators, physicians and patients were not blinded due to the study design.

### Intervention

In the intervention group, ED and intensive care physicians were asked to recommend a systematic ICU admission for all included patients. In case of unavailability of an ICU bed, another ICU bed had to be found in another or other hospital. In that case, patient’s transfer was done in priority in wards or centres participating in the study. If no ICU bed was available in participating wards/centres, patients could be transferred in a ward/centre not participating in the study. In these patients, the case report forms were completed as appropriate. All patients had to undergo a bedside evaluation by the intensive care attending physicians. The clinical case of all patients had to be systematically discussed between the ED and intensive care attending physicians. At the beginning of the trial, in-hospital meetings were organized with members of the scientific committee, ED and ICU staff to introduce and explain the trial. During the study period, there were monthly visits by clinical research nurses and ICU admission rates were presented during a twice-yearly investigators’ meetings and through newsletters.

In the control group, ED and intensive care physicians did not receive any recommendation regarding ICU admission (usual care). For all included patients (intervention and control groups), the final decision for ICU admission was made by the clinician at bedside and/or the patient or their surrogate decision-makers.

### Data collection

Screening forms of eligibility criteria were available at the emergency department of each participating hospital. A case report form (CRF) was filled out for each included patient by the ED and intensive care attending physicians. We collected the following data: age; sex; demographic and social characteristics; living place; clinical and biological evaluations to estimate the SAPS3 [24] (in the ED, one hour before admission to ICU or to another ward); prior comorbidities; ADL scale [21]; circumstances of the ED visit (day, time, availability of an ICU bed); referring physician; ED and intensive care physicians’ characteristics (age, gender, years of experience); physicians’ requests for an ICU admission; patient’s and family’s wishes about ICU admission; circumstances of the decision about ICU admission; final triage decision; characteristics of the hospital stay (admission and discharge dates and locations); survival status at ICU and hospital discharge. For patients admitted to the ICU, we collected performed invasive procedures, mechanical ventilation, vasoactive drugs administration, massive fluid resuscitation (defined as greater than half of an estimated body blood volume) and renal replacement therapy.

### Follow-up

Follow-up at 6 months was performed through phone calls or written questionnaires. If the patient could not be reached, relatives and/or the general practitioner was contacted. When necessary, the vital status was retrieved from appropriate legal institutions.

### Conduct of the study

The clinical research unit facilitated the conduct of the trial through an effective logistical coordination:

- Study nurses helped gathering missing information from CRFs and made follow-up phone calls;
- Clinical research assistants were responsible for centres set-up, monthly visits to intervention centres, completeness audit, database management and centres closures.
- Weekly control visits were organized in centres with low recruitment rates.

### Outcome measures

The primary outcome was the overall mortality at 6 months (individual level outcome).

Secondary outcomes were:

- ICU admission rate,
- ICU mortality,
- Hospital mortality,
- Functional status at 6 months (as assessed by the ADL scale [21]),
- Quality of life at 6 months (as assessed by the SF-12 Health Survey [25]).

A substudy of caregivers’ burden of care with only two centres is also planned. The evaluation of the burden of care was performed using the Zarit Burden Interview [26].

### Sample size and statistical analysis plan

#### Sample size

Using data from the ICE-CUB 1 study, we estimated a 32 % 6-month mortality rate in the control group. Considering an estimated intracluster correlation coefficient of 0.01, we estimated that 3000 patients would provide a power of 74 % to detect a difference of 6 % in mortality rates between the two groups, with a two-sided type 1 error rate of 0.05. We planned to recruit 25 centres during an inclusion period of 2 years and a half, based on a predicted average of 56 included patients per year per centre (data from ICE-CUB 1 study).

#### Statistical analysis plan

We will perform an intention-to-treat analysis using multilevel and mixed models. Specifically, random effect models will be used to take into account the clustered nature of the data. Multilevel logistic models with robust variance will be used for binary outcome variables and mixed effect Cox model for survival data. We planned a subgroup analysis on patients admitted in the ICU and with at least one organ support (mechanical ventilation, renal replacement therapy or vasopressors). R (The R Foundation for Statistical Computing, Vienna, Austria) will be used for statistical analyses. No interim analysis is planned.

### Quality of the data

The quality of the data was assessed by an independent clinical research assistant through data monitoring online. Systematic tests for consistency of the data were performed. Five per cent of the CRFs were randomly analysed. If discrepancies were >5 % in a centre, all data registered for that centre were verified.

### Ethical considerations and legal requirements

The study protocol was approved by the institutional review board (Comité de Protection des Personnes Ile de France), and all responsible authorities from each centres provided consent. Patient’s or surrogate decision-makers’ non-opposition to trial participation was assessed. An information sheet with contact details was handed to all patients and/or surrogate decision-makers. The authorization to use the patient’s data could be withdrawn by the patient or the surrogate decision-makers at any time.

The nominative database was approved by CCTIRS and CNIL (reference # 911503). The study was registered on Clinical trials.gov (NCT01508819). The electronic case report form (eCRF) was developed by URC-Ouest (TS) and URC-Est (SA), Paris, using online system CleanWeb (http://www.tentelemed.com/en/cleanweb/).

The scientific committee was composed of scientists and physicians from different specialties and backgrounds: intensive care medicine (BG, MG), emergency medicine (DP), geriatric medicine (CT), statistics (AB) and research unit of the hospital (TS).

### Funding

The study was funded by the French ministry of health (PHRC 2010 AOM 10154 K100103). The funding source had no interference with the conduct of the study. The research sponsor was the DRCD Ile-de-France who served as an independent Data Safety Monitoring Board (DSMB) (Project Code: K100103/No. IDRCB 2011-A00758-33). The DSMB had full access to the mortality data and could stop the trial in case of important disparity in mortality between groups. None of the members of the scientific committee nor the investigators declared any conflict of interest related to this study.

##  Results

**Fig. 1:**
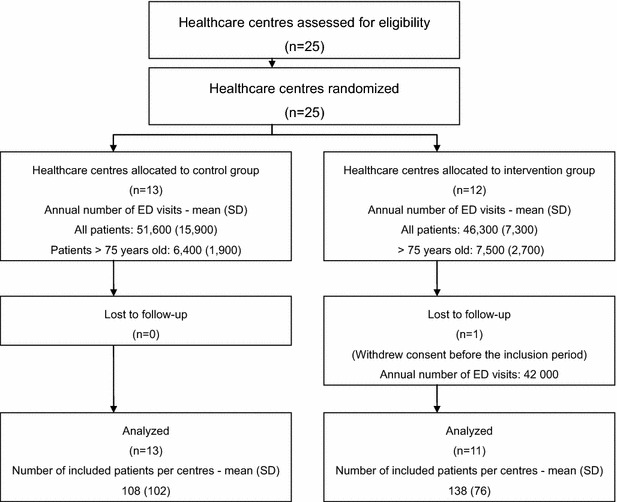
Flow chart

**Fig. 2:**
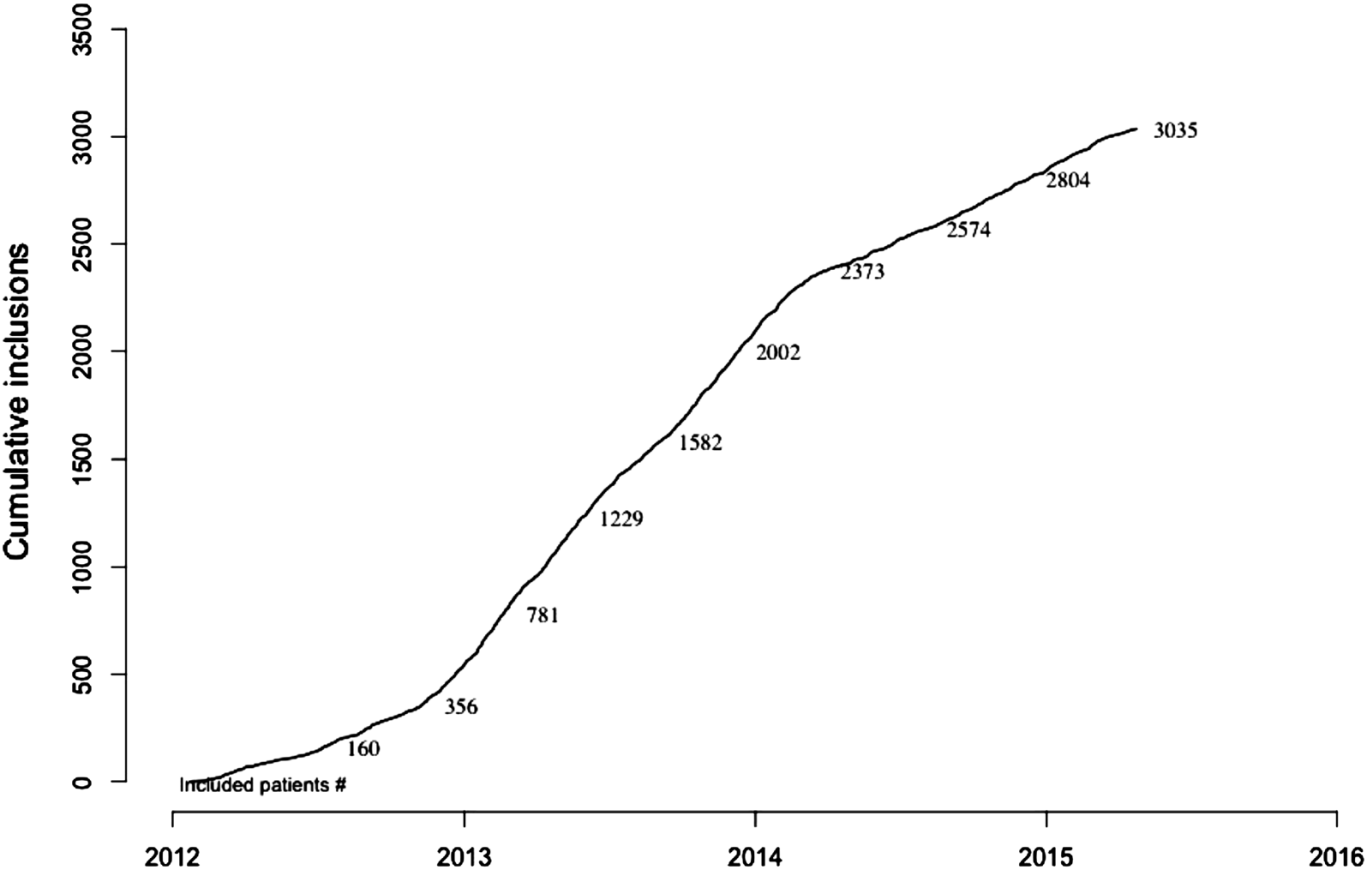
Inclusion curve of study patients. Date of inclusion is missing for one patient

Twenty-five healthcare centres were randomized to the intervention or the control group (Figs. 1, 2). One centre withdrew consent to participate after the randomization, leaving 24 centres participating to the study. Eleven centres were allocated to intervention group, and 13 centres were allocated to standard care. There were 18 academic centres and 6 non-academic centres. Fifteen centres were located in the Paris area and 9 in other areas in France. Between January 2012 and April 2015, 3036 patients were included in the trial (1518 patients in the intervention group and 1518 patients in the control group). One patient withdrew consent. There were 51 protocol violations for 49 patients (Table 2). Missing values were rare (Table 3). The ADL scale was completed for 83 % of patients.

**Table 2:** Number of protocol violations by categories

Protocol violations	Standard care (*n* = 1518)	Intervention group (*n* = 1518)
ADL score <4 or not evaluable	17	5
Age <75 years	5	1
Presence of cachexia	2	5
Absence of a pre-established critical conditions	4	4
No social security coverage	3	1
Presence of an active cancer	3	1
Total of protocol violations	33^a^	16^a^

^a^One patient did not meet two inclusion criteria in each group

**Table 3:** Main variables of the case report form

Item	% missing
Inclusion criteria	
Katz Index of Independence in Activities of Daily Living	16.63
Age at inclusion	0.00
Preserved nutritional status (defined as the absence of cachexia, subjectively assessed by physician at bedside)	0.00
Main reason for ED visit	0.13
Social security coverage	0.00
No known active cancer	0.00
Main variables	
Date of inclusion	0.03
SAPS III	6.03
Gender	0.00
Referring emergency physician’s seniority	1.61
Referring ICU physician’s seniority	33.86
Full ICU	41.63
Primary referent	6.52
Identified general practitioner	1.15
Invalidating illness	2.37
Living place	1.05
Home support	5.90
Physicians sought patient’s opinion regarding ICU admission	40.74
Patient’s opinion about ICU admission	0.10
Physicians seek primary referent’s/family’s opinion regarding ICU admission	49.47
Primary referent’s/family’s opinion	0.23
Perceived burden for primary referent/family	63.24
Surgical status at ED visit	0.30
Glasgow Coma Scale	12.02
Emergency physician proposal for an ICU admission	3.29
Reason for not proposing ICU	22.69
Patient wishes about ICU admission	42.42
ICU physician proposal for an ICU admission	20.55
Reason for not proposing ICU	4.42

## Discussion

This cluster-randomized clinical trial will assess the benefit of a strategy of recommendation for systematic ICU admission for critically ill elderly patients who get to the emergency department. Our trial was successfully completed and could overcome methodological, ethical and practical issues. Recruitment period lasted 36 months instead of the planned 30 months due to a lower than expected recruitment rate. There were few missing data.

Several constraints made this trial difficult to design and implement. First, despite the clinical equipoise about ICU admission for critically ill elderly patients [10, 18], randomization at the individual patient level was deemed unethical by virtue of beneficence, non-maleficence and respect of the patient’s autonomy. To overcome this barrier, we designed a cluster-randomized trial of a strategy of recommendation for systematic ICU admission for critically ill elderly patients. As the control group was assigned to standard practices regarding ICU admission, patients were not exposed to additional risks than usual. Second, a refusal of ICU admission for elderly patients may consist in a limitation of life-sustaining therapy and refers to an end-of-life decision-making process. The cluster design facilitated the feasibility of the study, as no treatment limitation was imposed to any patient in the course of the study. Furthermore, the final decision for ICU admission was made by the clinician at bedside and/or the patient or their surrogate decision-makers. Third, recruitment period lasted longer than expected due to a lower recruitment rate. Several actions had to be implemented to keep the hospital staff motivated owing to a longer than expected recruitment period. Fourth, elderly patients may have several disabilities (deafness, memory and cognitive impairment, comprehension difficulties) which complicated the follow-up. Finally, the study relied on a shared-decision model between the ED and the intensive care physicians in the intervention arm. We had to make efforts to foster the implication of both ED and ICU teams during the implementation of the study (Additional file 1).

## Conclusion

The ICE-CUB 2 trial was deemed feasible and ethically acceptable. This study will be the first cluster-randomized clinical trial to assess the benefit on long-term outcomes of a recommendation for systematic ICU admission for selected critically ill elderly patients.

## Additional file

10.1186/s13613-016-0161-5 Members of the ICE-CUB 2 study network.
